# Efficacy and Safety of the Collagenase of the Bacterium Clostridium Histolyticum for the Treatment of Capsular Contracture after Silicone Implants: Ex-Vivo Study on Human Tissue

**DOI:** 10.1371/journal.pone.0156428

**Published:** 2016-05-27

**Authors:** Sebastian Fischer, Christoph Hirche, Yannick Diehm, Kristo Nuutila, Jurij Kiefer, Emre Gazyakan, Ericka M. Bueno, Thomas Kremer, Ulrich Kneser, Bohdan Pomahac

**Affiliations:** 1 Department of Surgery, Division of Plastic Surgery, Brigham and Women’s Hospital, Harvard Medical School, Boston, Massachusetts, United States of America; 2 Department of Hand-, Plastic and Reconstructive Surgery, Burn Trauma Center, BG Trauma Center Ludwigshafen, University of Heidelberg, Ludwigshafen, Germany; University of Akron, UNITED STATES

## Abstract

**Background:**

The fibrotic capsule that surrounds silicone implants consists mainly of collagen. The FDA-approved collagenase of the bacterium clostridium histolyticum provides a reasonable treatment option. Safety and efficacy at the female breast site must be evaluated before clinical utilization.

**Materials and Methods:**

We incubated 20 samples of fibrotic capsule as well as 12 full thickness skin grafts harvested from the female breast site for 24 hours with different doses of collagenase. Outcome measures involved histological assessment of thickness and density of the capsule tissue as well as the skin grafts. Furthermore, we performed a collagen assay and immunohistochemistry staining for collagen subtypes.

**Results:**

Collagenase treatment was able to degrade human capsule contracture tissue ex-vivo. The remaining collagen subtype after degradation was type 4 only. 0.3 mg/ml of collagenase was most effective in reducing capsule thickness when compared with higher concentrations. Of note, effectiveness was inversely related to capsule density, such that there was less reduction in thickness with higher capsule densities and vice versa. Furthermore, the application of 0.3mg/ml collagenase did not lead to thinning or perforation of full thickness skin grafts.

**Conclusion:**

Adjustment of collagenase dose will depend on thickness and density of the contracted capsule. A concentration of 0.3mg/ml seems to be safe and effective in an ex-vivo setting. The remaining collagen subtype 4 is suitable to serve as a neo-capsule/acellular tissue matrix. Collagenase treatment for capsular contracture may soon become a clinical reality.

## Introduction

Capsular contracture is the most common long-term complication after silicone-based breast augmentation. Incidence ranges from 5 to 55% depending on study type and follow-up period [1–5]. To date, the only established therapy is surgical incision or resection of the fibrous capsule, which involves the removal of the implant in most cases [6]. Non-surgical approaches to overcome capsular contracture have failed due to lack of efficiency or unbearable side effects [7].

According to current literature, development of capsular contracture is based on subclinical infection due to biofilm formation on the implants surface and foreign body reaction to the silicone material itself, among others [8–10]. Both mechanisms maintain a chronic inflammatory response, which stimulates fibroblast proliferation and differentiation into myofibroblasts. The latter have contractile properties and are capable of an excessive synthesis of collagen, which is the main component of the fibrous envelope.

The collagenase of the bacterium clostridium histolyticum, commercially available as Xiaflex, digests every type of collagen with the exception of collagen type 4 [11]. The latter is an important component of vessel walls, perineurium and the basal lamina, and thus needs to be preserved for safe clinical application. Xiaflex is approved for the treatment of Dupuytren’s contracture and Peyronie’s disease and reported side effects are low in incidence and severity [12, 13].

The first attempts to utilize Xiaflex for treatment of capsular contracture in a rodent model were recently reported by our group; the results were encouraging regarding applicability and effectiveness [14]. Local injection of collagenase led to dosage-depending degradation of fibrotic tissue which was evident in histology and magnetic resonance imaging. However, almost 20% of animals suffered skin perforation at the implant site. Skin perforation would be a major limitation for clinical application. The reasons or mechanisms leading to skin perforation are not completely understood yet. There may be direct digestion of the skin by Xiaflex, resulting in weakened skin structure. In addition there may be a mechanical component to skin perforation, due to the animal’s movements. Consequently, we assume that the concentration of Xiaflex used in those animals that sustained skin perforation was exceedingly high, and further dose adjustment studies are necessary. Of note, rat skin is three times thinner and 10 times more permeable compared with human skin, making comparison of dose and effectiveness of the collagenase difficult [15, 16].

Therefore, the aim of this study was to investigate the efficacy of Xiaflex on human capsule tissue samples as a function of capsule thickness, collagen density, and concentration as well as safety regarding perforation of skin harvested from the female breast site.

## Materials and Methods

### Human tissue samples

Discarded human breast skin and capsule contracture tissue samples were collected anonymously. For anonymously collected discarded tissue samples Institutional Review Board approval is not required. Breast skin was gathered during breast reduction or breast lift surgery in female patients suffering from pathologically enlarged breasts or ptosis. Of note, breast skin samples included the full thickness skin graft only, thus without subcutaneous tissue or breast-related adipose tissue, mammary gland or muscle. Capsule contracture tissue was collected during capsulectomies in female patients suffering from capsular contracture grade 2 to 4 according to Baker classification.

### Collagenase

The collagenase of the bacterium clostridium histolyticum produced and provided by Auxilium Pharmaceutical, Inc. (Xiaflex, Horsham, PA, USA) was used in all experiments in this study. This drug consists of powder and solvent for solution.

### Incubation Breast Skin and Capsule Tissue Samples

**Fig 1:**
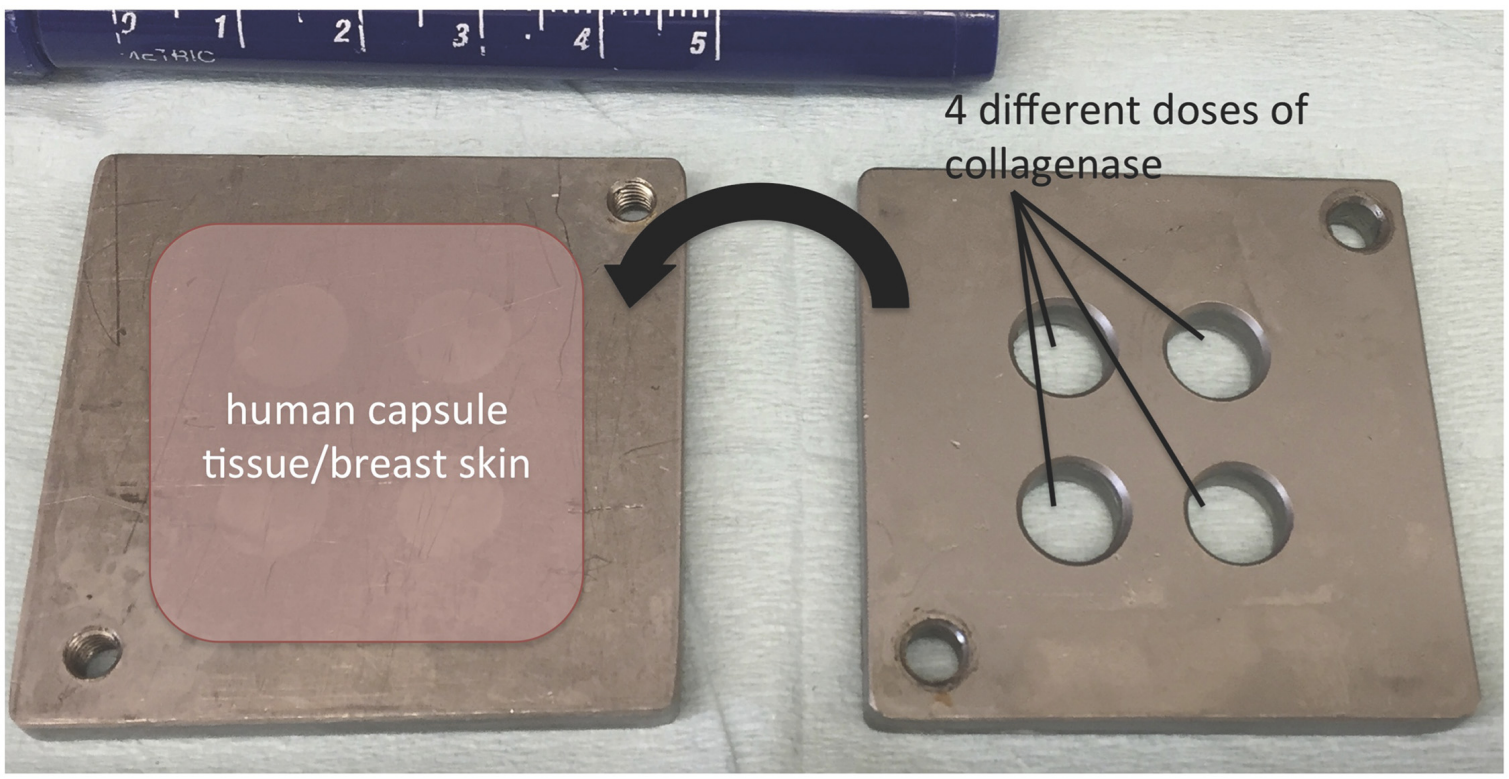
Custom-made titanium chamber for incubation of capsular contracture tissue and full thickness breast skin samples with 4 different doses of collagenase.

Breast skin (n = 12) and capsule tissue (n = 20) samples were cut into 5 cm × 5 cm pieces and mounted into a custom-made titanium device with 4 isolated chambers, each of which allowed application of collagenase at a different concentration (Fig 1). In addition, an extra piece of capsule tissue was cut into 4 equally sized cubes of 0.4 cubic centimeters each, which were further transferred into four 2.5 ml Eppendorf tubes containing collagenase. Test concentrations of collagenase in the 4 chambers and 4 tubes were 0.3mg/ml, 0.9mg/ml and 1.8mg/ml, as well as solvent only as a control. Samples were incubated for 24 hours and subsequently prepared for histology or collagen assays. According to the manufacturer the collagenase becomes inactivated within 12 hours after in-vivo application. Therefore, incubation periods longer than 24 hours were not anticipated.

### Histology

Samples were fixed in formalin and embedded in paraffin. Hematoxylin eosin staining and Masson tri-chrome staining were utilized for assessment of the skin and capsule thickness, respectively. The entire thickness of the skin and capsule samples was measured using ImageJ (v. 1.46, NIH, USA) at 10 randomly chosen sites by two blinded investigators.

#### Collagen Density

Picro Sirius red staining under light microscope enabled for precise identification of collagen fibers in capsule tissue and skin samples. Assessment of density was performed with ImageJ (v.1.46, NIH, USA) at 10 randomly selected sites chosen by two blinded investigators and results are given in percentage of the area of the analyzed image.

#### Collagen subtype analysis

To distinguish between the different collagen types within capsule tissue samples, we analyzed the above-mentioned Picro Sirius red staining with a circularly polarized light microscope (LV100N POL, Nikon, Japan) for collagens type 1 and 3 [17, 18], and performed immunohistochemistry staining to identify collagens 2 and 4. The latter involved monoclonal antibodies (Anti-Collagen II antibody, 2 μg/ml ab185430; Anti-Collagen IV, 1/500, ab6586; Abcam, Cambridge, MA, USA) used according to manufacturer’s recommendation. Briefly, formalin-fixed, paraffin-embedded sections were de-paraffinized and a heat-mediated antigen retrieval was performed. Following incubation with primary and secondary antibodies, diaminobenzidine (SK-4105, Vector Laboratories Burlingame, CA, USA) was used as chromogen and nuclei were counterstained with hematoxylin dye. Lastly, slides were dehydrated and cover-slipped.

### Collagen Assay

For collagen quantification, the Sircol Soluble Collagen Assay Kit (Biocolor, Carrickfergus, Northern Ireland) was used according to the manufacturer’s instructions. The tissue samples were weighed and subsequently incubated in 0.5M acetic acid with 0.1mg/ml pepsin overnight to isolate the collagen. Subsequently, Sircol Dye Reagent was added to precipitate and stain the collagen. After washing with Acid-Salt Wash Reagent the collagen pellets were re-suspended in 250μl Alkali Reagent and absorbance was measured at 555nm using a spectrophotometer (SpectraMax Plus384, Molecular Devices, Sunnyvale, CA, USA). Collagen concentrations were calculated using a standard curve generated according to the manual and given in μg collagen per gram tissue.

### Statistical analysis

Student t-test for paired samples was used for statistical analysis of capsule and skin thickness, collagen density and collagen content. A p-value of <0.05 was considered as statistical significant.

## Results

### Capsule Thickness

**Fig 2:**
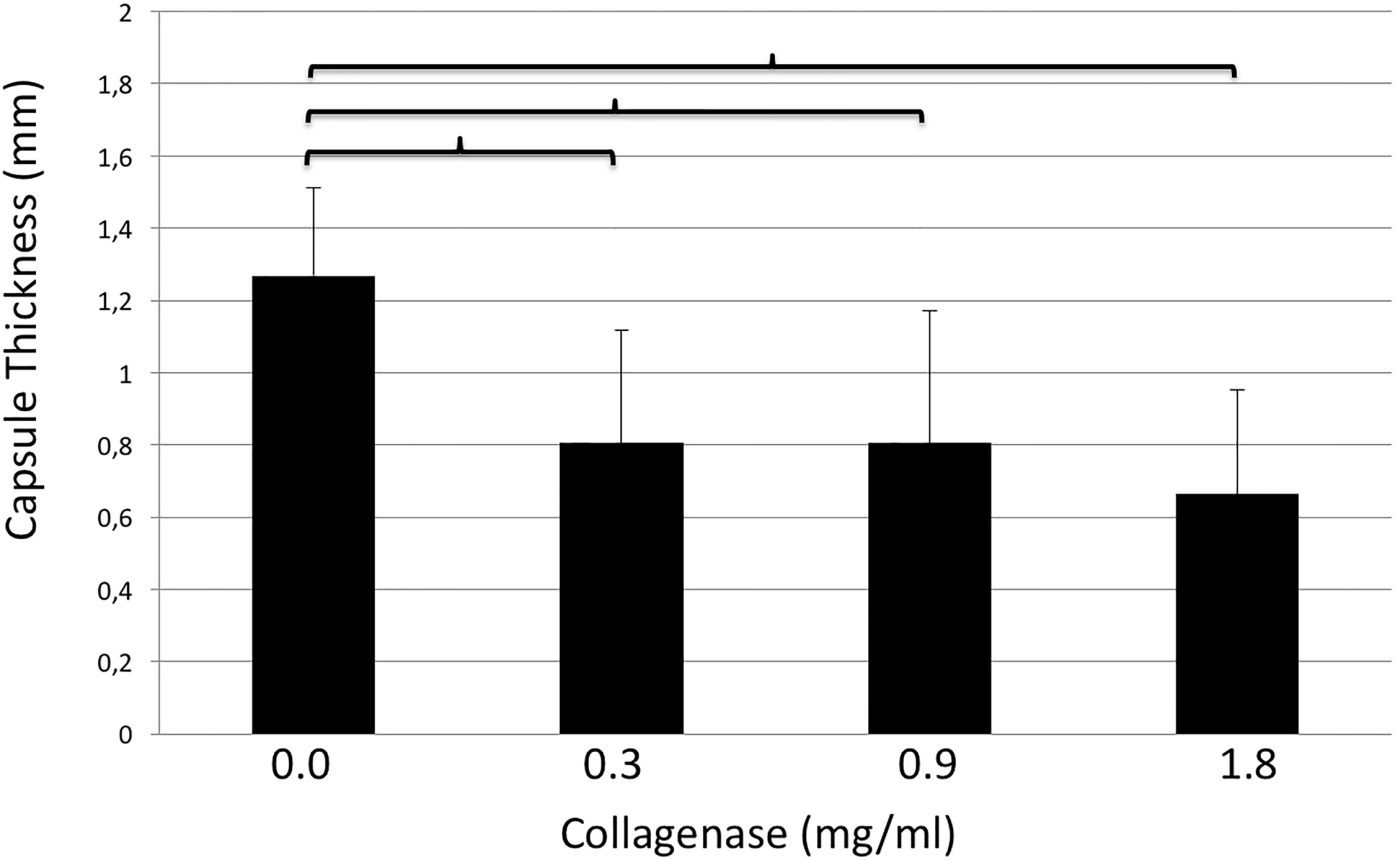
Thickness of capsule contracture tissue samples (y-axis, mm) after incubation with different doses of collagenase (x-axis, mg/ml). Brackets indicate statistically significant differences (p<0.05).

**Fig 3:**
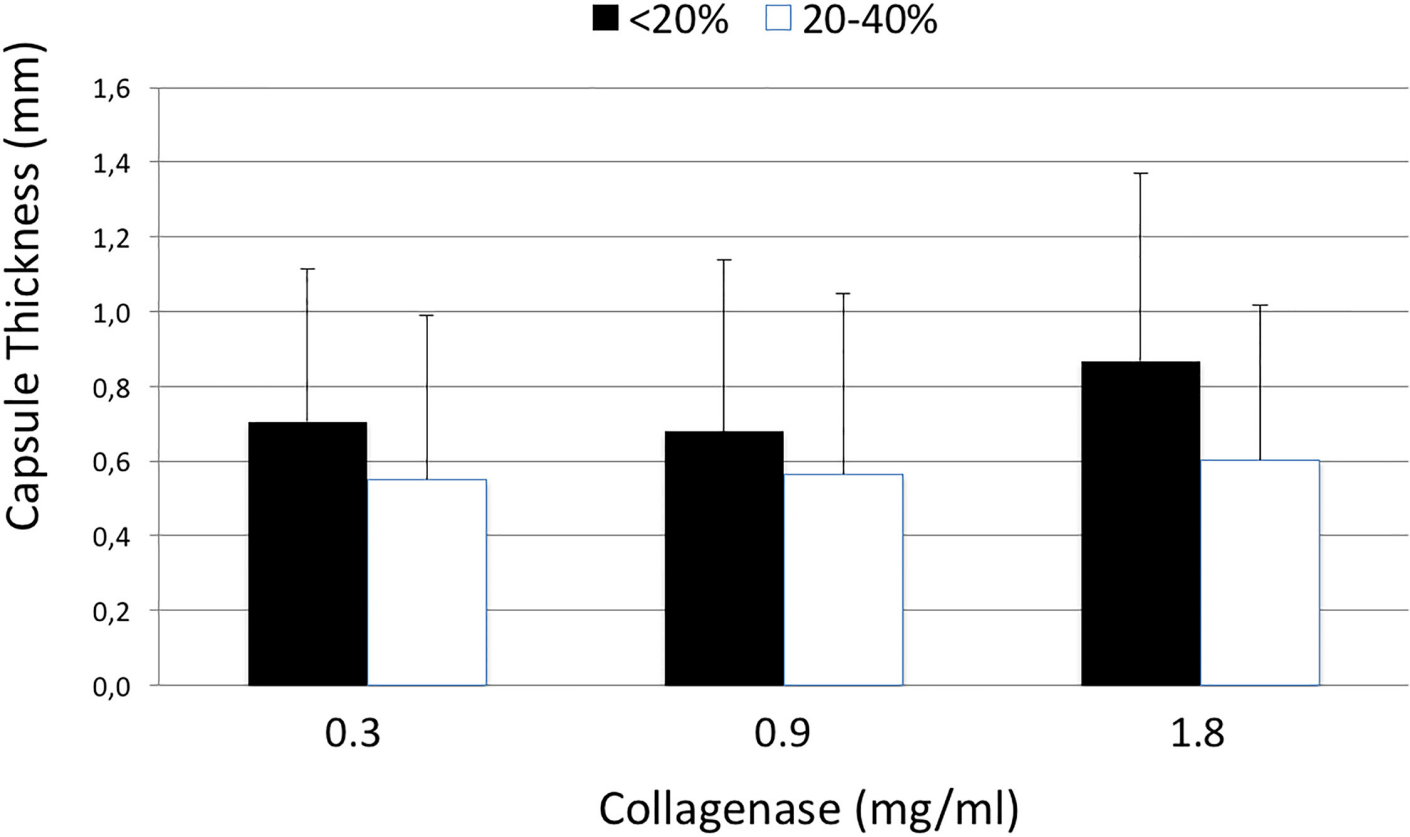
Thickness of capsule contracture tissue samples (y-axis, mm) after incubation with different doses of collagenase (x-axis, mg/ml) in correlation to capsule density (<20%, black and 20–40%, white).

The thicknesses of capsule tissues after incubation with 0.0, 0.3, 0.9, and 1.8mg/ml of collagenase were 1.27±0.2, 0.8±0.3, 0.8±0.4 and 0.7±0.3mm, respectively. The thickness of control samples was significantly higher than every other group (0.0 vs. 0.3mg/ml, p = <0.001; 0.0 vs. 0.9mg/ml, p = 0.001; 0.0 vs. 1.8mg/ml, p = <0.001). Results are shown in Fig 2. These groups were further subdivided according to collagen density into two subgroups, namely <20% (Subgroup A) and 20–40% (Subgroup B). Reductions in capsule thickness after collagenase treatment when compared with control groups were as follows: Subgroup A (<20% collagen density): CG-0.3mg/ml: 0.71±0.41mm; CG-0.9mg/ml: 0.67±0.46mm and CG-1.8mg/ml: 0.86±0.5mm; Subgroup B (20–40% collagen density): CG-0.3mg/ml: 0.55±0.44mm; CG-0.9mgml: 0.56±0.48mm and CG-1.8mg/ml: 0.6±0.41mm. Results are depicted in Fig 3.

### Capsule Density

**Fig 4:**
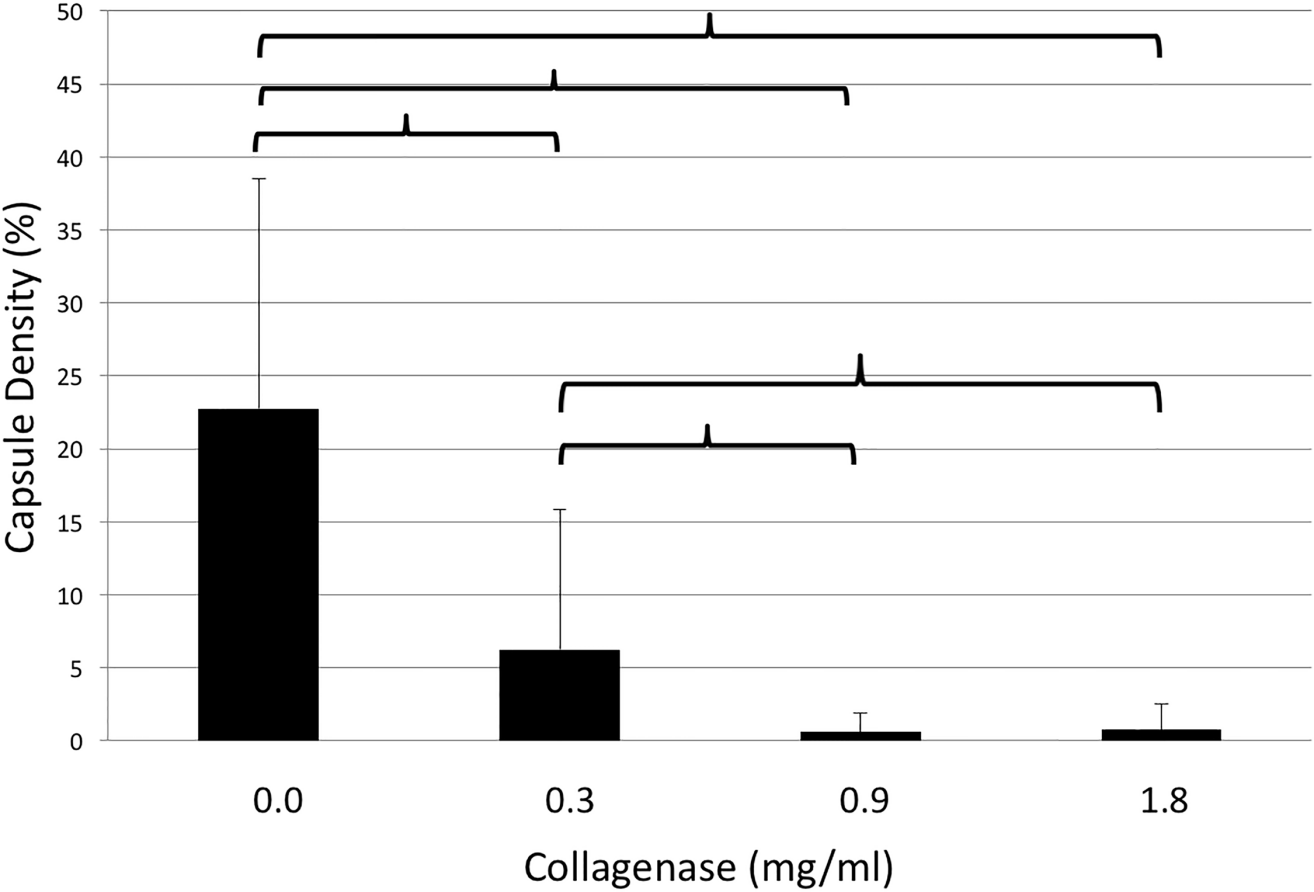
Density of capsule contracture tissue samples (y-axis, %) after incubation with different doses of collagenase (x-axis, mg/ml). Brackets indicate statistically significant differences (p<0.05).

The density of capsules was 22.8±15.7, 6.2±9.6, 0.6±1.3, and 0.7±1.7% after incubation with 0.0, 0.3, 0.9, and 1.8mg/ml of collagenase, respectively. Therefore, all study concentrations led to significant decreases in capsular collagen density when compared with the control group (0.0 vs. 0.3mg/ml, p = 0.002; 0.0 vs. 0.9mg/ml, p<0.001; 0.0 vs. 1.8mg/ml, p<0.001). Furthermore, treatment with 0.3mg/ml collagenase lead to a significantly higher decrease in capsule thickness when compared with both higher concentrations (0.3 vs. 0.9mg/ml, p = 0.041; 0.3 vs. 1.8mg/ml, p = 0.046). Density measurements are demonstrated in Fig 4.

### Collagen concentration

**Fig 5:**
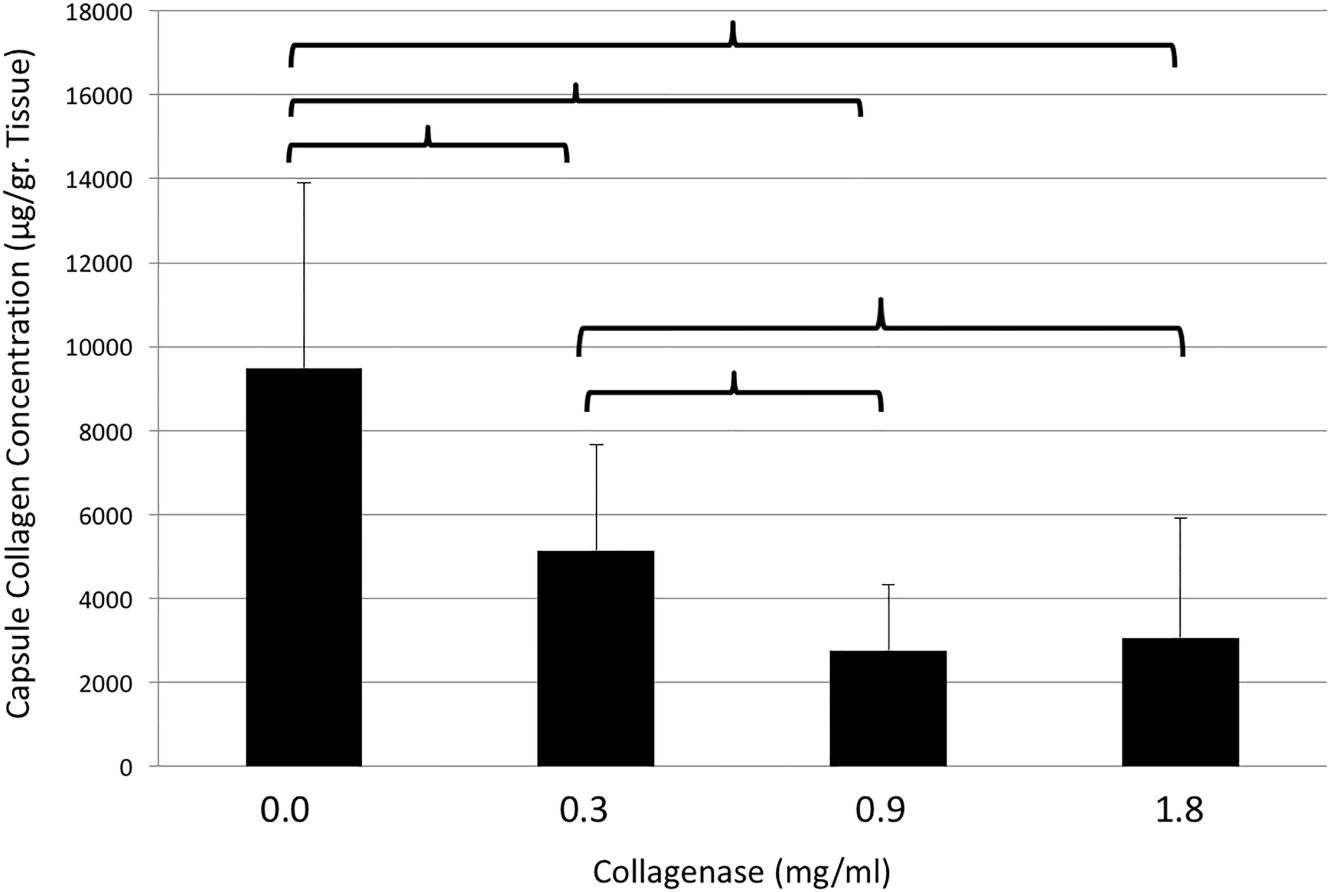
Collagen concentration of capsule contracture tissue samples (y-axis, μg/gr. Tissue) after incubation with different doses of collagenase (x-axis, mg/ml). Brackets indicate statistically significant differences (p<0.05).

After incubation with 0.0, 0.3, 0.9, and 1.8mg/ml of collagenase, collagen concentrations were 9498.6±4404.2, 5150.9±2528.3, 2760.7±1568, and 3069.8±2847.6 μg/gr, respectively. Compared to the control group, samples in each treatment group showed a significant decrease in collagen concentration (0.0 vs. 0.3mg/ml, p = 0.006; 0.0 vs. 0.9mg/ml, p<0.001; 0.0 vs. 1.8mg/ml, p<0.001). Furthermore, a dosage depending effect was evident within study groups, with significant decrease in collagen concentration after incubation with 0.9mg/ml and 1.8mg/ml when compared with 0.3mg/ml (p = 0.011 and p = 0.049, respectively). There were no significant differences detected in the collagen concentration of samples treated with 0.9 and 1.8mg/ml collagenase (p>0.05). Results are shown in Fig 5.

### Collagen subtype analysis

**Fig 6:**
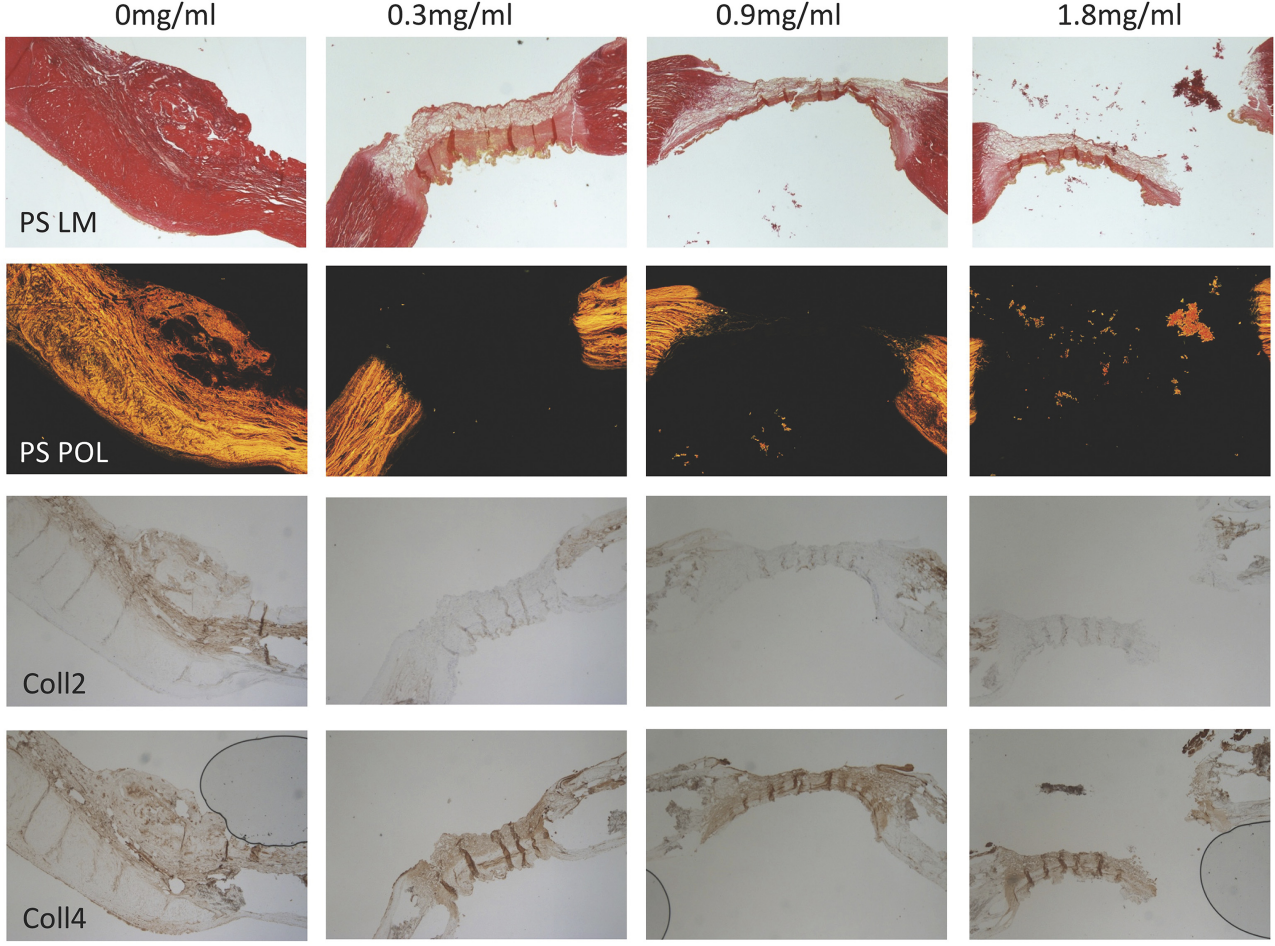
Example pictures of Picro Sirius red staining under light microscopy, circularly polarized light microscopy and immunohistochemistry staining for collagen 2 and 4 (from top to bottom) of capsule contracture tissue after incubation with 0.0, 0.3, 0.9, and 1.8mg/ml of collagenase (from left to right). After collagenase incubation only collagen 4 is detectable in immunohistology all other collagen subtypes were successfully digested.

Picro Sirius red and antibody staining for collagens 1 to 4 demonstrated that after incubation with collagenase, the remaining tissue consisted of collagen type 4 only. Fig 6 shows examples of capsule tissue stained with Picro Sirius red and antibodies against collagen 2 and 4 after incubation with different doses of collagenase.

### Skin thickness

**Fig 7:**
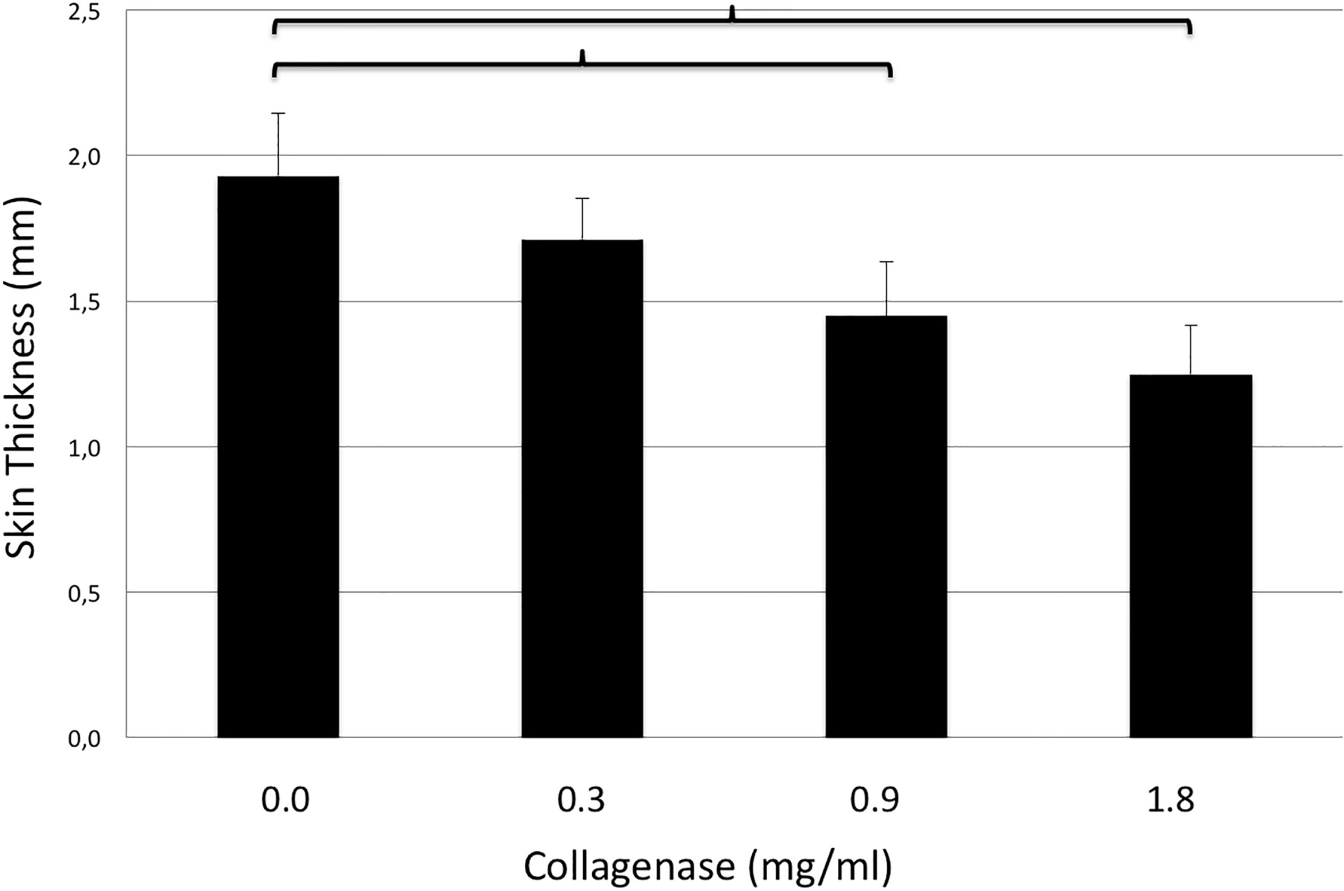
Thickness of full-thickness breast skin grafts (y-axis, mm) after incubation with different doses of collagenase (x-axis, mg/ml). Brackets indicate statistically significant differences (p<0.05).

**Fig 8:**
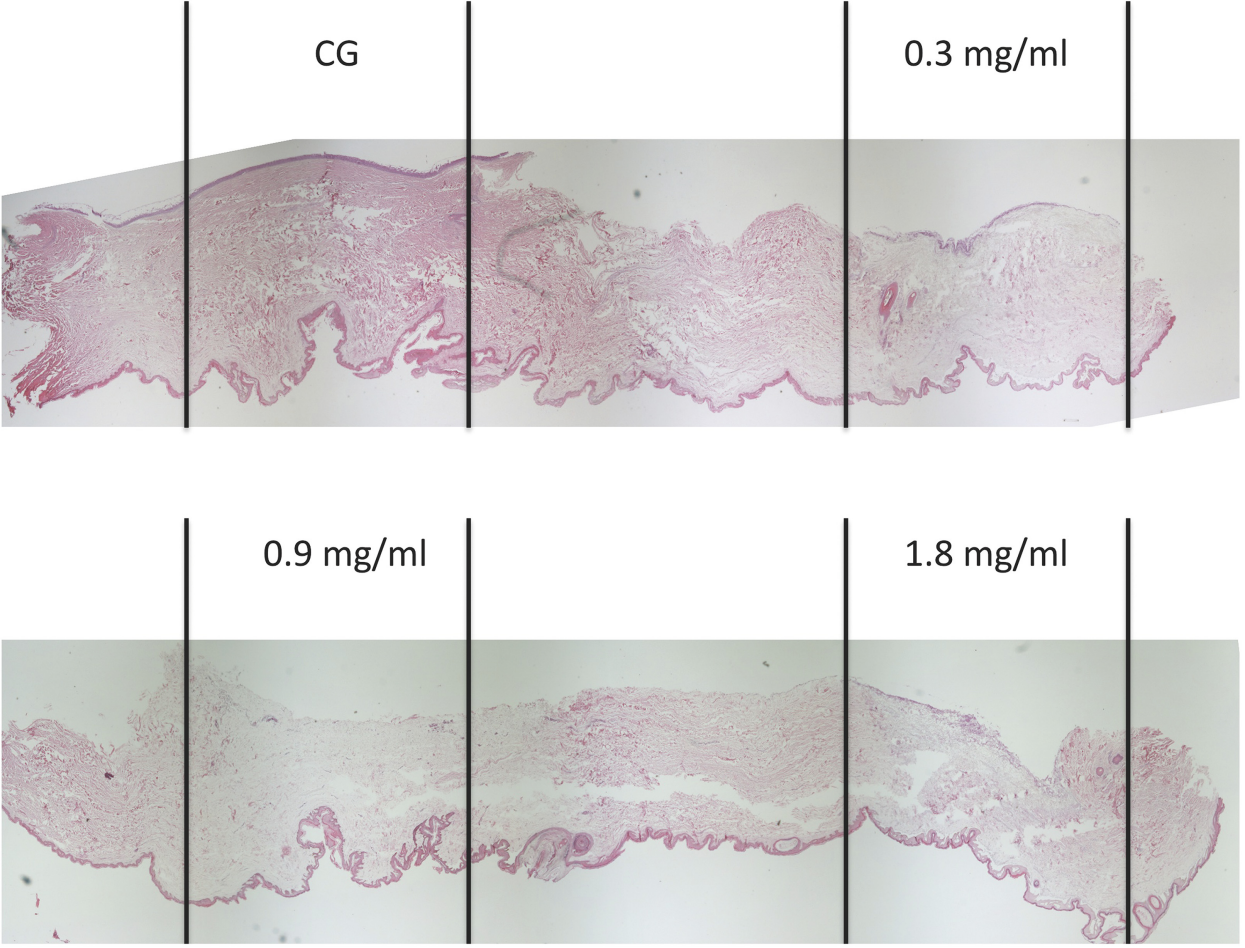
Example pictures of HE staining under light microscopy of full thickness breast skin grafts after incubation with 0.0 and 0.3mg/ml of collagenase (top from left to right) and 0.9, and 1.8mg/ml (bottom from left to right). While 0.9 and 1.8mg/ml of collagenase lead to significant thinning of the skin compared to control, 0.3mg/ml did not lead to significant differences. Importantly, skin perforation did not occur in any sample.

After 24 hours of incubation with collagenase, skin thickness was significantly different between the control group (CG: 1.93±0.22mm) and both higher concentrations, namely 0.9 and 1.8mg/ml (0.9mg/ml: 1.45±0.18mm; p = 0.038; 1.8mg/ml: 1.25±0.17mm; p = 0.021). There were no differences in skin thickness detected between the control group and 0.3mg/ml (0.3mg/ml: 1.71±0.14mm, p<0.05), nor within study groups (Fig 7). Importantly, skin perforation did not occur in any sample. A sample skin section is shown in Fig 8.

### Skin density

**Fig 9:**
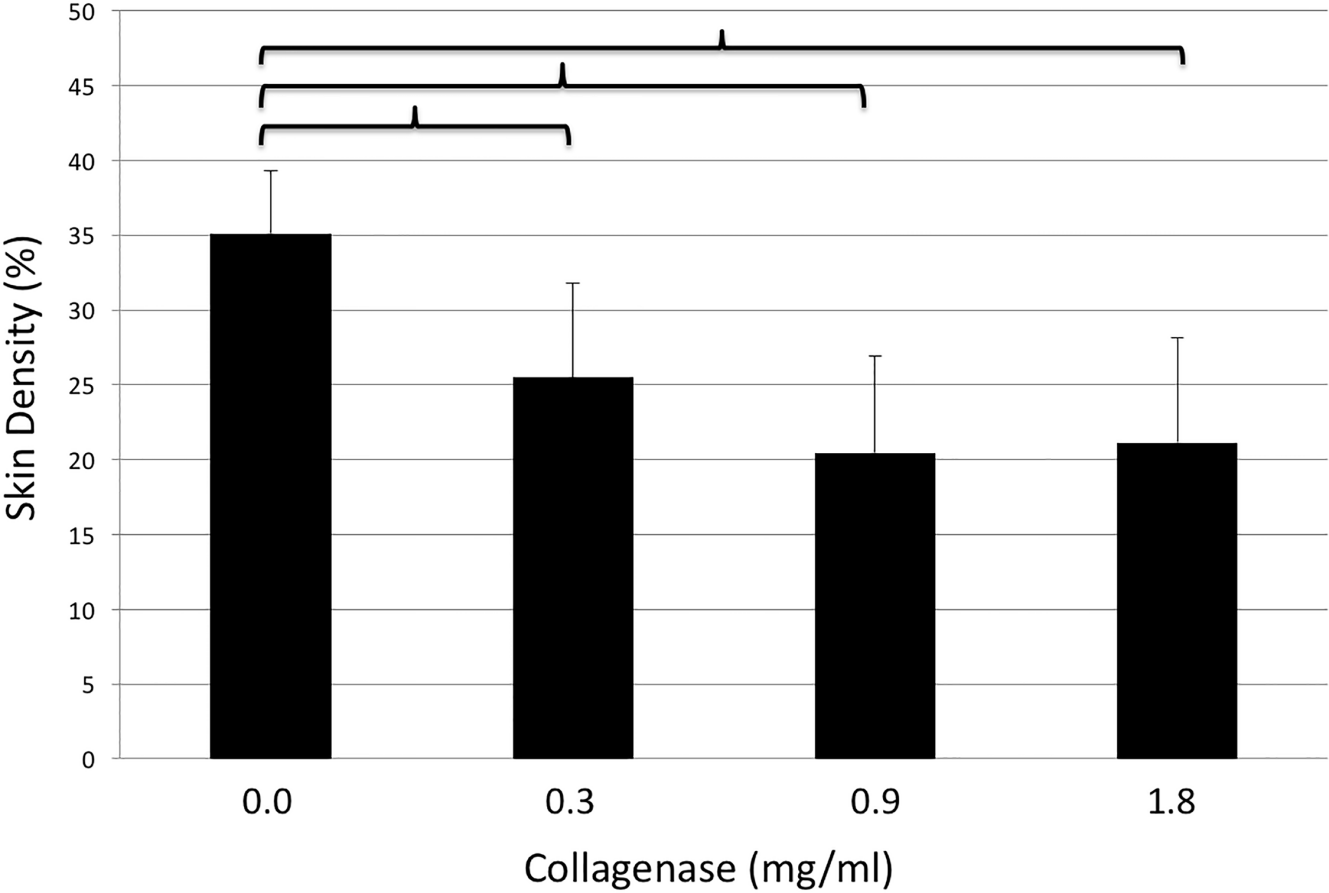
Density of full-thickness breast skin grafts (y-axis, %) after incubation with different doses of collagenase (x-axis, mg/ml). Brackets indicate statistically significant differences (p<0.05).

The collagen density of full thickness skin grafts was 35.1±4.2, 25.5±6.3, 20.4±6.5 and 21.1±7% after incubation with 0.0, 0.3, 0.9 and 1.8mg/ml, respectively. Significant differences in skin density were detected between the control group and each of the study groups (0.0 vs. 0.3mg/ml, p = 0.044; 0.0 vs. 0.9mg/ml, p = 0.031; 0.0 vs. 1.8mg/ml, p = 0.03). Collagen density did not differ significantly among study groups. Results are summarized in Fig 9.

## Discussion

In this study, we demonstrated that the collagenase of the bacterium clostridium histolyticum is capable of degrading human capsule contracture tissue ex-vivo. Collagenase at a dose of 0.3 mg/ml was most effective at reducing capsule thickness when compared with higher doses. Of note, effectiveness was dependent on the density of the capsule itself, with improved thickness reduction for lower density capsules and vice versa. Furthermore, the application of collagenase did not lead to perforation of full thickness skin grafts harvested from the female breast site, and thus has the potential for safe clinical application for treatment of capsular contracture after silicone based breast augmentation.

Several studies in the current literature address the development of non-surgical treatment options for capsular contracture after silicone implants [19, 20]. However, only a few studies involve human tissue or even clinical trials. In 1991, Spear et al. injected steroids into double lumen mammary prostheses to prevent capsular contracture in patients undergoing immediate breast reconstruction after mastectomy. Capsule contracture rates were 14% at 3 months and remained on this level for the following 3 years. In contrast, a pair-matched control group, which received saline filled double lumen implants without steroids, revealed a contracture rate of 44% after the same study period. These findings were substantiated by Lemperle et al. who reported a decrease in contracture rates from 64% to 24% due to preventive administration of prednisone into double lumen implants.

Despite these promising results, steroids did not become the golden standard for prevention of capsular contracture. Related complications prohibited broad application of steroid filled implants. The risk of skin thinning and perforation as well as dislocation of the implant below the infra-mammary fold, did not overweight the risk of capsular contracture occurrence [21].

In our study we saw a decrease in skin thickness depending on the collagenase concentration. Skin thinning became statistically significant with concentrations of 0.9mg/ml and higher. In contrast, skin density was significantly lower compared with the control, irrespective of the applied concentration and without differences among study groups. Importantly, skin perforation was not seen in any sample. With respect to clinical application, collagenase was incubated in direct contact with the inner side of human full thickness skin grafts, thus missing out subcutaneous fat tissue and capsule tissue, which would provide additional barriers for skin protection in-vivo. Nevertheless, our own group has observed that in-vitro results of collagenase effects on skin can change dramatically in an in-vivo setting in a rodent model of capsular fibrosis [14]. While skin perforation was absent in-vitro, incidence was almost 20% after in-vivo application. The latter was explained by mechanical irritation of the weakened skin by the textured silicone implant. While the implant was localized above the scapula, it scratched continuously at the inside of the skin during each movement of the rat, which eventually led to perforation. In our opinion, mechanical irritation is well controllable in a clinical setting. Temporary fixation of the breast with means of bras or bandages as well as physical rest will significantly reduce movements and provide stabilization until the collagenase gets inactivated. Of note, the latter occurs within 8 hours due to serum proteins or even immediately after oral intake of tetracycline antibiotics. The ability of spontaneous or induced inactivation provides a major advantage towards steroid filled breast implants. While the continuous release of steroids led to chronic skin thinning and lowering of the infra-mammary fold, inactivation of collagenase will allow for reformation of a neo-capsule and thus tissue stabilization after degradation or significant weakening of the contracted capsule.

Another non-surgical approach to overcome capsular contracture, but this one with better controllability than steroids was followed by Reid et al. [22]. The authors administered Zafirlukast, which is FDA-approved for asthma treatment, to women suffering from capsular contracture. Zafirlukast reveals anti-inflammatory capacities and is capable of inhibiting collagen synthesis as well as contraction of myofibroblasts. After 3 months of treatment, the authors revealed a significant decrease of the initial Baker grade in 75% of cases including complete conversion to Baker grade 1 in more than two-thirds of the study population. These findings were substantiated in a prospective study of Scuderi et al., who applied Zafirlukast to 120 patients with capsular contracture and revealed a decrease of capsule hardness by 24% in average after 6 months of treatment [23]. Despite these encouraging results, long-term studies demonstrated that softening of the capsule is maintained only during Zafirlukast treatment, leading to immediate increase of hardness after withdrawal [24]. The latter, however, is necessary, as chronic abuse of Zafirlukast can cause hypertension and severe liver damage [25].

In this context, Peimer et al. recently published first long-term results of collagenase treatment for Dupuytren’s contracture [13]. Whereas treatment-related adverse events were mild to moderate in all cases, overall recurrence rates were 47% after 5 years and thus comparable to surgical treatment. Of note, in case of recurrence, repeated collagenase injection was again an effective treatment option. With respect to capsular contracture, these results are difficult to apply, as the silicone implant provides an on-going stimulus for the fibrotic reaction. However, our own group was able to show persistence of thinner capsules after in-vivo injection of collagenase in a rodent model of capsular fibrosis (submitted). Histology as well as non-invasive in-vivo imaging demonstrated significantly thinner capsules compared to control groups 2 months after collagenase application. In addition, gene analysis revealed a significantly lower expression of TGFbeta3, which is a pro-fibrotic marker, thus indicating less fibrotic activity in the long run. Taking into account that 2 months in rodents equate to 6 years in humans, these results are promising for long-term effectiveness of collagenase treatment of capsular fibrosis [26]. Long-term persistence of collagen reduction was further substantiated by Syed et al., who demonstrated that the collagenase utilized in our studies is capable for down-regulation of extra cellular matrix components, cytokines and growth factors, leading to an inhibition of fibroblast migration and proliferation [27]. Nevertheless, it is currently unclear if collagenase treatment for capsular contracture will lead to a persistent reduction of capsule tissue in humans. Degradation of the collagenous capsule will bring the implant surface in direct contact with underlying structures that can trigger an inflammatory process and may result in an early and more severe capsule formation.

In general, doubts associated with collagenase treatment for capsular contracture are not related to effectiveness rather than safety and longevity of the effect. As the fibrotic capsule that surrounds the silicone implant consists mainly of collagen, collagenase application is potentially the most reasonable treatment option currently available. Accordingly, we were able to demonstrate that collagenase treatment leads to a significant decrease in thickness, density and collagen concentration of human capsule tissue after ex-vivo incubation. This effect was based on collagen degradation with selective preservation of collagen subtype 4, as proven by immunohistochemistry staining. Importantly, efficacy in reducing capsule thickness was dependent on the density of the capsule tissue. To achieve the same reduction in thickness, capsules with low collagen density required lower concentrations of collagenase and vice versa. The latter gains importance in the light of dose adjustment of collagenase to prevent side effects.

Ideally, the administered collagenase partially weakens rather than completely digests the contracted capsule. Subsequently, relief of tension can be obtained with means of manual compression in a 24-hour interval. This approach of combined enzymatic and manual capsulotomy will warrant the security as surrounding structures will not get in contact with the active enzyme. However, to bring this approach into practice it is necessary to find (1) an optimum dose that does not completely perforate the capsule and (2) an application technique that allows for minimally invasive administration of the collagenase into the space between implant and capsule. While the former might be possible with means of latest imaging technology facilitating dose adjustment to capsule thickness and density of each individual patient, the latter would require modifications of silicone implants or novel medical devices. In the meantime, and as ideal circumstances are always challenging to achieve, the applied concentration of collagenase should be as low as damage of surrounding tissue is unlikely. Therefore, our study provides the first benchmark, namely 0.3mg/ml of collagenase, which is capable of significant degradation of capsule tissue without leading to skin thinning or perforation in an ex-vivo setting.

## Conclusion

The collagenase of the bacterium clostridium histolyticum was capable of degrading human capsule contracture tissue in a dose dependent manner. Effectiveness at decreasing capsule thickness depended on the density of the capsule itself. Furthermore, in this ex-vivo approach, 0.3mg/ml of collagenase did not thin out full thickness skin grafts harvested form the female breast site. Collagenase treatment for capsular contracture in terms of an enzymatic capsulotomy has the potential to become soon clinical reality.
